# Microsomal glutathione transferase 1 confers cisplatin resistance of non-small cell lung cancer via interaction with arachidonate lipoxygenase 5 to repress ferroptosis

**DOI:** 10.22038/ijbms.2024.79203.17160

**Published:** 2025

**Authors:** Jun Yuan, Rui Zhang, Li Liu, Yue-song Ban, Ce Qin

**Affiliations:** 1 Department of Thoracic Surgery, Cangzhou Central Hospital, Cangzhou061000, Hebei Province, P.R. China; 2 Department of Oncology, North China Petroleum General Hospital, Cangzhou062550, Hebei Province, P.R. China; 3 Department of Thoracic Surgery, Cangzhou People’s Hospital, Cangzhou061000, Hebei Province, P.R. China; 4 Department of Thoracic Surgery, Huanghua People’s Hospital, Cangzhou061100, Hebei Province, P.R. China; 5 Department of Oncology, Cangzhou Central Hospital, Cangzhou061000, Hebei Province, P.R. China

**Keywords:** Acetylsalicylic acid, Antioxidants, Epididymis, Melatonin, Sperm, Testosterone

## Abstract

**Objective(s):**

Cisplatin (DDP) resistance remains a primary cause of chemotherapy failure and recurrence of non-small cell lung cancer (NSCLC). Abnormal high microsomal glutathione transferase 1 (MGST1) expression has been found in DDP-resistant NSCLC cells. This study aimed to explore the function and mechanism of MGST1 in DDP resistance of NSCLC cells.

**Materials and Methods:**

The expression levels of target molecules were assessed by quantitative real-time polymerase chain reaction (RT-qPCR) and western blotting. Cell proliferation was evaluated by cell counting kit-8 (CCK-8) and colony formation assays. Ferroptosis was determined by malondialdehyde (MDA), glutathione (GSH), Fe^2+^, and reactive oxygen species (ROS) levels. The interaction between proteins was confirmed by Co-immunoprecipitation (Co-IP). The effect of MGST1 on DDP resistance was evaluated using the tumor xenograft assay in vivo. Immunohistochemical staining was performed to measure Ki-67 and p-H2A.X expression in tumor tissues.

**Results:**

MGST1 expression was higher, while arachidonate lipoxygenase 5 (ALOX5) expression was lower in DDP-resistant NSCLC patients and cells. *MGST1* ablation sensitized NSCLC cells to DDP therapy through inducing ferroptosis. MGST1 protein directly interacted with ALOX5 protein to restrain ALOX5-triggered ferroptosis. Ferroptosis inhibitor or sh-ALOX5 reversed the promotive effect of MGST1 silencing on the DDP sensitivity of NSCLC cells. Finally, *MGST1* depletion sensitized NSCLC cells to DDP therapy in nude mice *in vivo*.

**Conclusion:**

MGST1 high expression contributed to DDP resistance of NSCLC cells by inhibiting ALOX5-induced ferroptosis. Our results provide a potential therapeutic target for overcoming DDP resistance in NSCLC patients.

## Introduction

Lung cancer is a common malignancy with poor outcomes, high occurrence, and mortality (1). As one of the important subtypes, non-small cell lung cancer (NSCLC) accounts for about 85% of all lung cancer patients (2). Currently, cisplatin DDP (cisplatin) chemotherapy is considered the first-line therapy for advanced NSCLC in the clinic (3). However, the severe side effects and development of drug resistance greatly limit the therapeutic efficacy of DDP (4). Therefore, there is an urgent need to elucidate the possible mechanisms of DDP resistance and develop an effective therapeutic strategy to improve the outcomes of NSCLC patients.

Ferroptosis is one of the crucial types of programmed cell death caused by iron-dependent lipid peroxidation (5). Recently, the involvement of ferroptosis in the development and chemotherapy resistance of NSCLC cells has been documented. For example, Deng *et al*. reported that Spectrin Beta, a Non-Erythrocytic 2 (SPTBN2) inhibitor, overcame the DDP resistance of NSCLC cells through ferroptosis induction (6). Notably, DDP has been demonstrated to induce ferroptosis via depleting GSH (7). Therefore, ferroptosis induction is recognized as an effective strategy to overcome the DDP resistance of NSCLC cells. However, the detailed regulatory mechanisms of ferroptosis have not been fully explored. 

Microsomal glutathione transferase 1 (MGST1) belongs to the MAPEG family, a membrane-bound enzyme with anti-oxidative capacities (8). Aberrant high expression of MGST1 has been reported in multiple types of cancers, including lung cancer (9). Mounting evidence has suggested that MGST1 exerts an inhibitory effect on ferroptosis in tumor cells, which might be a promising therapeutic target (10). A previous study has demonstrated that MGST1 was up-regulated in DDP-resistant NSCLC cells (11). So far, the influence of MGST1 on the DDP resistance of NSCLC cells remains largely unknown. Arachidonate lipoxygenase 5 (ALOX5) is a member of the lipoxygenase family that plays an important role in catalyzing various lipid compounds (12). Previous studies have documented that dysregulation of ALOX5 was correlated with ferroptosis resistance in various cancers (13, 14). Low expression of ALOX5 has been identified to have a close association with immune cell infiltration in NSCLC (15). Of note, it has been reported that MGST1 directly interplayed with ALOX5 to cause ferroptosis inhibition via decreasing lipid peroxide production in pancreatic cancer cells (16). Thus, we predicted that MGST1 might confer resistance of NSCLC cells to DDP via regulation of ALOX5-mediated ferroptosis escape. This study aimed to verify this speculation, and our findings demonstrated that MGST1 was highly expressed and ALOX5 was lowly expressed in DDP-resistant NSCLC tissues and cells. Depletion of MGST1-induced ferroptosis to enhance DDP sensitivity of NSCLC cells through direct interaction with ALOX5. Therefore, we suggest that MGST1 inhibition might be a promising strategy to overcome DDP resistance in NSCLC.

## Materials

***Clinical sample collection***

NSCLC and para-carcinoma samples were collected from 30 NSCLC patients through surgical operation at Cangzhou Central Hospital. According to the response to DDP treatment, the NSCLC patients were divided into two groups: the DDP-resistant group (n=19) and the DDP-sensitive group (n=11). All NSCLC patients provided their informed consent. The ethics committee of Cangzhou Central Hospital approved the experiment procedures.

***Cell culture, establishment of DDP-resistant cells, and treatment***

A549 and NCI-H1975 cells were purchased from the American Type Culture Collection (ATCC, USA). A549 cells were cultured with F-12K (Gibco, USA), and NCI-H1975 cells were cultured with RPMI-1640 Medium (Gibco, USA) containing 10% fetal bovine serum (Gibco) at 37 ^°^C with 5% CO_2_. DDP-resistant cells were established by treatment with increasing DDP concentrations as previously described (17). In brief, A549 and NCI-H1975 cells were treated with 0.5 μg/ml DDP (MCE, USA) for 72 hr. After recovery for 72 hr, the cells were treated with gradually increasing doses of DDP (1, 2, 4, 8, and 10 μg/ml). After the above treatments for three cycles, DDP-resistant cells (named A549/DDP and NCI-H1975/DDP) were successfully established. To inhibit ferroptosis, the A549/DDP and NCI-H1975/DDP cells were treated with the ferroptosis inhibitor Ferrostatin-1 (10 μM, MCE) for 24 hr. 

***Cell transfection***

Short hairpin RNA targeting *MGST1* (sh-*MGST1*), sh-*ALOX5*, and negative control shRNA (sh-NC) were obtained from GenePharma (Shanghai, China). A549/DDP and NCI-H1975/DDP cells were transfected with sh-*MGST1*, sh-*ALOX5*, or sh-NC using Lipofectamine 2000 (Thermo Fisher, USA). The stably transfected cells were selected by treatment with decreasing doses of Neomycin (800 to 200 μg/ml) for 14 days.

***Quantitative real-time polymerase chain reaction (RT-qPCR)***

Total RNA was extracted from NSCLC tissues and cells using the TRIzol reagent (Thermo Fisher), followed by cDNA synthesis using the RT Master Mix for qPCR II (MCE). The mRNA levels of *MGST1* and *ALOX5 *were detected using the SYBR Green Premix Ex Taq ROX plus (Takara, Japan) on the CFX96 Touch Real-Time PCR Detection System (Bio-Rad, USA). The relative mRNA levels were calculated using the 2^−ΔΔCT^ method. The primer sequences are as follows: 

*MGST1*, forward: GCCAATCCAGAAGACTGTGTAGC, reverse: AGGAGGCCAATTCCAAGAAATG

G; *ALOX5*, forward: CCTATGCCTCCCTGTGCT, 

reverse: TGGTCGCCCTCGTAG

TAGA; *β-Actin*, forward: TTGCGTTACACCCTTTCTTG, reverse: CACCTTCACCGTTCCAGTTT.

***Western blotting***

Protein samples were isolated from NSCLC cells using the RIPA buffer (Beyotime, Haimen, China). The protein concentration was determined using the BCA protein quantification kit (Yeasen, Shanghai, China). The protein samples (40 μg) were separated by sodium dodecyl sulfate-polyacrylamide gel electrophoresis (SDS-PAGE), followed by blotting onto polyvinylidene fluoride (PVDF) membranes. The membranes were blocked with 5% skim milk for one hour and then incubated with primary antibodies against MGST1 (PA5-79670, 1:1000, Thermo Fisher), ALOX5 (#3289, 1:1000, CST, USA), glutathione peroxidase4 ( GPX4, A21440, 1:500, ABclonal, Wuhan, China), solute carrier family 7 member 11 (SLC7A11, A2413, 1:500, ABclonal), Acyl-CoA synthetase (ACSL4, A20414, 1:1000, ABclonal), and reduced glyceraldehyde-phosphate dehydrogenase (GAPDH, A19056, 1:10000, ABclonal) overnight at 4 ^°^C, followed by incubation with horseradish peroxidase-conjugated secondary antibody (AS014, 1:2000, ABclonal) for one hour. The protein bands were visualized using the ECL western Blotting Substrate (Solarbio, Beijing, China).

***Cell counting kit-8 (CCK-8)***

CCK-8 assay was performed to determine cell viability. Briefly, A549/DDP and NCI-H1975/DDP cells were seeded into 96-well plates ( 3×10^3 ^cells per well). After various transfections, 10 µl of CCK8 reagent (Beyotime) was added into each well, followed by incubation for two hours at 37 °C. Finally, the results were measured at OD450 on a microplate reader (Bio-Tek, USA). 

***Colony formation***

DDP-resistant NSCLC cells were seeded into 6-well plates (1000 cells per well). After two weeks of maintenance in the culture medium, the cells were fixed with 4% paraformaldehyde and subsequently stained with crystal violet dye for 20 min. The colonies were photographed and quantified. 

***Detection of malondialdehyde (MDA), glutathione (GSH), Fe*** ^2+^ ***, and reactive oxygen species (ROS) levels***

The MDA, GSH, Fe^2+^, and ROS levels were detected using the commercial MDA Content Assay Kit, GSH Content Assay Kit, Cell Iron Content Assay Kit, and ROS Assay Kit provided by Solarbio, following the manufacturer’s protocols. 

***Co-immunoprecipitation (Co-IP)***

DDP-resistant NSCLC cells were lysed with RIPA lysis buffer (Beyotime) and centrifugated at 12,000 g for 10 min. Cell lysates were collected and pre-cleared with protein A agarose beads at 4 ^°^C for three hours. Subsequently, cell lysates were immunoprecipitated with anti-ALOX5 (#3289, CST) or anti-IgG (AC042, ABclonal) in the presence of protein A agarose beads at 4 ^°^C overnight with rotation. Then, the immunoprecipitated proteins were eluted from protein A agarose beads and detected by western blotting.

***Animal model***

Four-week-old male BALB/c nude mice were purchased from SLAC Laboratory Animal Co. Ltd (Shanghai, China). There were four experimental groups (n=6 per group): control, DDP, DDP+sh-NC, and DDP+sh-*MGST1*. A549/DDP cells (3×10^6^) with or without stable transfection with sh-NC or sh-*DHCR24 *were subcutaneously injected into the left flank of mice. When the tumor diameter reached about 5 mm, 4 mg/kg, DDP was intraperitoneally injected into the mice every three days, six times in total (18). The tumor volume was evaluated every three days using the formula: volume= 1/2×width × length^2^. All mice were euthanized by cervical dislocation at the end of the experiment. The xenografts were collected and weighed. All animal experiments were approved by the Committee on the Ethics of Cangzhou Central Hospital (Ethical code: 2022-187-03(z)).

***Immunohistochemical staining***

The xenografts were subjected to fixation with 4% paraformaldehyde, dehydration, and embedding in paraffin. Subsequently, the paraffin-embedded tumor tissues were cut into 4-μm sections. After dewaxing and antigen repair, the sections were incubated with antibodies against Ki-67 (#34330, 1:200, CST) or p-H2A.X (AP0687, 1:50, ABclonal) at 4°C overnight and then incubated with secondary antibody (AS014, 1:2000, ABclonal) for two hours. The sections were developed with 3,3′-diaminobenzidine staining and photographed under light microscopy (Olympus, Japan).

***Statistical analysis***

Data are shown as mean±standard deviation (SD). GraphPad Prism 6.0 was used for statistical analysis using Student’s t-test between two groups or one-way analysis of variance (ANOVA) for multiple groups. Pearson correlation analysis was adopted to assess the correlation between *MGST1* and *ALOX5* mRNA expression. A *P*-value less than 0.05 was considered statistically significant. 

**Figure 1:**
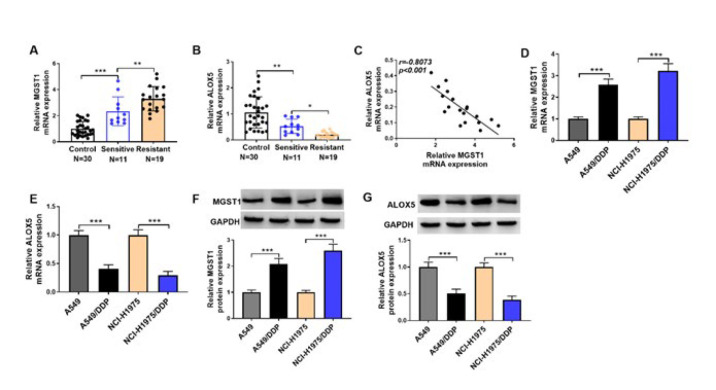
MGST1 was up-regulated, but ALOX5 was down-regulated in DDP-resistant NSCLC samples and cells

RT-qPCR analysis of *MGST1* (A) and *ALOX5* (B) mRNA levels in non-cancerous tissues, DDP-resistant and sensitive NSCLC tissue. (C) Correlation between *MGST1* and *ALOX5* mRNA levels in NSCLC samples was assessed by Spearman correlation analysis. *MGST1* (D) and *ALOX5 *(E) mRNA levels in DDP-resistant NSCLC cells were determined by RT-qPCR. Western blotting analysis of MGST1 (D) and ALOX5 (E) protein abundance in DDP-resistant NSCLC cells. Data are expressed as mean±SD. * *P<*0.05, ** *P<*0.01, *** *P<*0.001

**Figure 2:**
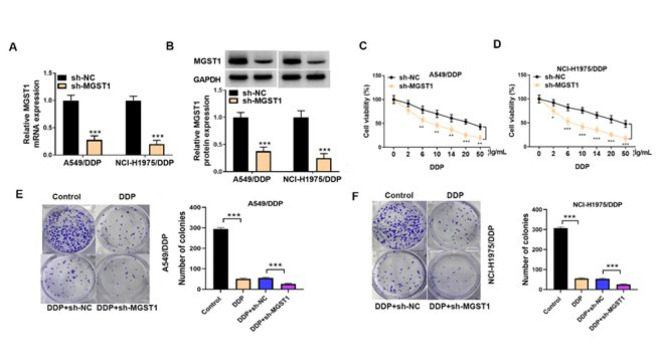
MGST1 knockdown sensitized NSCLC cells to DDP therapy

A549/DDP and NCI-H1975/DDP cells were transfected with sh-*MGST*1 or sh-NC. (A)&(B) RT-qPCR and western blotting detected MGST1 mRNA and protein levels. (C)&(D) Cell viability of sh-*MGST1* or sh-NC-transfected cells was measured by CCK-8 after exposure to various concentrations of DDP. (E)&(F) The growth of NSCLC cells was determined by colony formation assay. Data are expressed as mean±SD. * *P<*0.05, ** *P<*0.01, *** *P<*0.001

**Figure 3:**
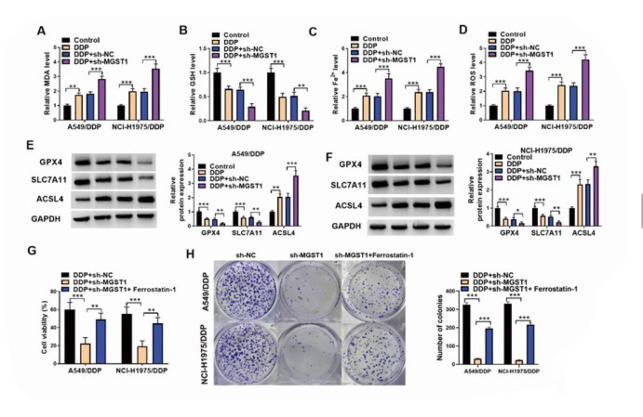
MGST1 silencing induced ferroptosis to elevate DDP sensitivity of NSCLC cells

A549/DDP and NCI-H1975/DDP cells were transfected with sh-*MGST1* or sh-NC in the presence of 10 μg/ml DDP. (A-D) The MDA, GSH, Fe^2+^, and ROS levels were measured by commercial kits. (E)&(F) The protein levels of GPX4, LC7A11, and ACSL4 were evaluated by western blotting. Sh-*MGST1*-transfected A549/DDP and NCI-H1975/DDP cells were treated with 10 μM Ferrostatin-1 in the presence of DDP. (G) Cell viability of DDP-resistant NSCLC cells was detected by CCK-8. (H) The growth ability was assessed by colony formation assay. Data are expressed as mean±SD. * *P<*0.05, ** *P<*0.01, *** *P<*0.001

**Figure 4:**
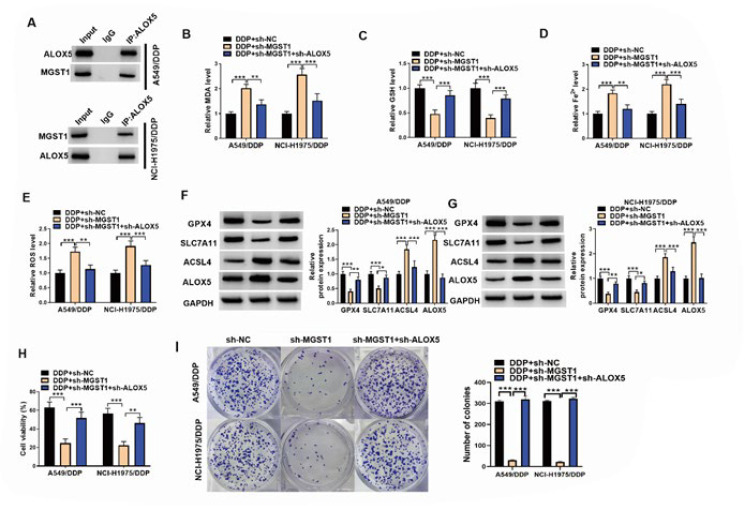
MGST1 led to ferroptosis inhibition in NSCLC cells via direct interaction with ALOX5

(A) Co-IP confirmed the interaction between MGST1 and ALOX5 proteins. Sh-*MGST1*-transfected DDP-resistant NSCLC cells were co-transfected with sh-*ALOX5* upon DDP exposure. (B-E) MDA, GSH, Fe^2+^, and ROS levels were assessed by commercial kits. (F)&(G) The protein abundance of GPX4, LC7A11, ALOX5, and ACSL4 was determined by western blotting. (H)&(I) Cell proliferation was determined by CCK-8 and colony formation assay. Data are expressed as mean±SD. * *P<*0.05, ** *P<*0.01, *** *P<*0.001

**Figure 5:**
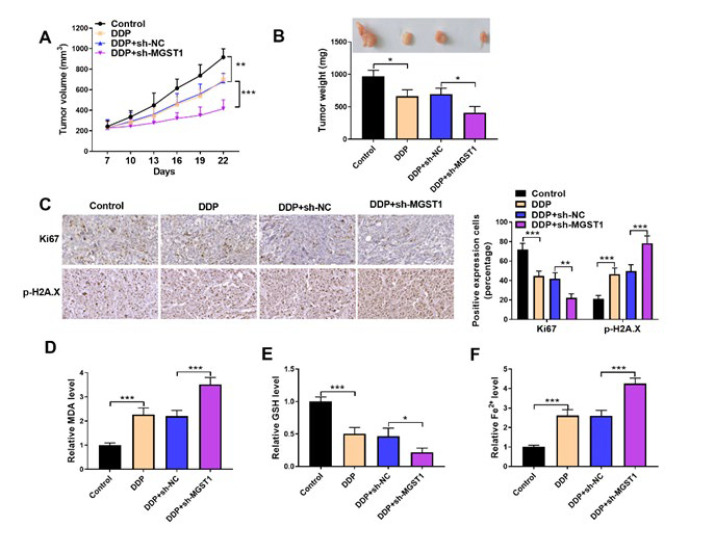
MGST1 silencing delayed xenograft growth and enhanced DDP sensitivity of NSCLC cells *in vivo*

The tumor volume (A) and weight (B) in different groups were measured. (C) Ki67 and p-H2A.X expression in the tumor tissues was evaluated by immunohistochemical staining. (D-F) MDA, GSH, and Fe^2+^ levels in the tumor tissues were assessed. Data are expressed as mean±SD. * *P<*0.05, ** *P<*0.01, *** *P<*0.001

## Results

***Up-regulation of MGST1, while down-regulation of ALOX5 in DDP-resistant NSCLC tissues and cells***

First, the abnormal expression of MGST1 and ALOX5 in the clinical samples of NSCLC was investigated. We found that *MGST1* mRNA was highly expressed, but *ALOX5* mRNA was lowly expressed in DDP-sensitive NSCLC tissues, and these changes were more evident in DDP-resistant NSCLC tissues (Figure 1A&B). In addition, there was a negative correlation between *MGST1* and *ALOX5* mRNA levels in NSCLC samples (Figure 1C). We further validated the above findings in A549/DDP and NCI-H1975/DDP cells *in vitro*. Consistent with* the vivo* data, both the mRNA and protein levels of MGST1 were higher in DDP-resistant NSCLC cells in comparison with the parental cells, while ALOX5 expression was lower in the DDP-resistance group (Figure 1D-G). Collectively, MGST1 was up-regulated, but ALOX5 was down-regulated in DDP-resistant NSCLC cells.

***MGST1 knockdown enhances the DDP sensitivity of NSCLC cells***

Given the abnormally high expression of MGST1 in DDP-resistant NSCLC cells, we further explored its biological function in this process. As shown in Figure 2A&B, MGST1 was silenced by stable transfection with sh-*MGST1* in A549/DDP and NCI-H1975/DDP cells. CCK-8 assay indicated that DDP-induced decreased viability of A549/DDP and NCI-H1975/DDP cells was evidently enhanced by MGST1 knockdown (Figure 2C&D). Moreover, DDP treatment decreased the number of colonies, which was further reduced after MGST1 depletion (Figure 2E&F). These results revealed that NSCLC cells were sensitized to DDP after MGST1 depletion. 

***Ferroptosis induction is involved in MGST1 silencing-mediated elevation in DDP sensitivity***

MGST1 has been reported to exert an inhibitory effect on ferroptosis in various cancer cells (19). Thus, we further investigated whether MGST1 reduced the DDP sensitivity of NSCLC cells by suppressing ferroptosis. The results showed that ferroptosis was induced in DDP-treated NSCLC cells, as confirmed by increasing MDA, Fe^2+^, and ROS levels and decreasing GSH levels (Figure 3A-D). As expected, MGST1 deficiency further raised MDA, Fe^2+^, and ROS levels and lowered the GSH level of NSCLC cells (Figure 3A-D). Additionally, we detected the changes in ferroptosis-related proteins. As assessed by western blotting, DDP treatment down-regulated GPX4 and SLC7A11 proteins and up-regulated ACSL4 protein, which could be intensified by MGST1 knockdown (Figure 3E&F). To further verify the involvement of ferroptosis in MGST1-mediated DDP resistance, sh-*MGST1*-transfected NSCLC cells were co-treated with ferroptosis inhibitor Ferrostatin-1. CCK-8 assay demonstrated that the decreased cell viability in sh-*MGST1*-transfected cells could be recovered by Ferrostatin-1 co-treatment in the presence of DDP (Figure 3G). Accordingly, Ferrostatin-1 co-treatment enhanced the reduced number of colonies caused by sh-*MGST1* (Figure 3H). Taken together, MGST1 silencing raised the DDP sensitivity of NSCLC cells via inducing ferroptosis.

***MGST1 directly interplays with ALOX5 to affect ferroptosis of NSCLC cells***

To further elucidate the possible modulatory mechanism of MGST1 in ferroptosis of NSCLC cells, we focused on ALOX5. Co-IP assay proved that MGST1 protein could directly interact with ALOX5 protein in A549/DDP and NCI-H1975/DDP cells (Figure 4A). To determine whether MGST1 modulated ferroptosis of NSCLC cells via regulation of ALOX5 expression, DDP-resistant NSCLC cells were transfected with sh-*MGST1* combined with or without sh-*ALOX5*. We found that the elevated MDA, Fe2+, and ROS levels and declined GSH levels in MGST1-silenced cells could be reversed by sh-*ALOX5* co-transfection (Figure 4B-E). Furthermore, ALOX5 ablation abolished MGST1 knockdown-induced down-regulation of GPX4 and SLC7A11 and up-regulation of ACSL4 in NSCLC cells (Figure 4F&G). In addition, ALOX5 knockdown counteracted the sh-*MGST1-*induced reduction in cell viability of DDP-resistant NSCLC cells (Figure 4H). Accordingly, the reduced number of colonies in the MGST1-depleted group was remarkably increased by ALOX5 down-regulation (Figure 4I). These findings suggested that MGST1 deficiency induced ferroptosis of NSCLC cells through interaction with ALOX5 protein.

***MGST1 deficiency restrains DDP resistance and tumor growth in vivo***

Finally, we investigated the regulation of MGST1 in DDP sensitivity in nude mice *in vivo*. DDP-induced reduction in tumor volume and weight was further strengthened by MGST1 depletion (Figure 5A&B). As determined by immunohistochemical staining, Ki-67 expression was decreased, while p-H2A.X expression was increased in DDP-treated tumor tissues, and these changes were further reinforced when MGST1 was silenced (Figure 5C). Besides, MGST1 down-regulation further enhanced DDP-induced elevation in MDA and Fe^2+^ levels and reduction in GSH concentration in tumor tissues (Figure 5D-F). Collectively, MGST1 deficiency triggered ferroptosis and enhanced DDP chemosensitivity in nude mice.

## Discussion

Chemotherapy resistance has been considered one of the major obstacles to the successful treatment of most cancers, including NSCLC (20). Multiple previous studies have suggested the involvement of differentially expressed genes or proteins in various disorders, including chemotherapy resistance of NSCLC cells (21-24). An earlier study reported the up-regulation of MGST1 in the development of NSCLC cells to DDP resistance (11); however, its potential regulatory mechanisms remain obscure. This study revealed that MGST1 expression was elevated in DDP-resistant NSCLC samples and cells. Functional experiments showed that MGST1 silencing remarkably increased DDP sensitivity and restrained malignant proliferation of DDP-resistant NSCLC cells. Mechanistically, MGST1 directly interacted with ALOX5 protein to repress ferroptosis of NSCLC cells, thereby leading to DDP resistance. Our results uncovered the biological functions and potential mechanisms of MGST1 in NSCLC cells suffering DDP resistance, providing an innovative way to conquer DDP-resistant NSCLC.

MGST1 is one of the Phase II detoxifying enzymes, which promotes cell survival by inhibiting the production of ROS (25). Dysregulation of MGST1 has been found in different human cancers (8, 26, 27). More importantly, the involvement of high expression of MGST1 in drug resistance of cancer cells has been confirmed. Precious studies have shown that MGST1 was up-regulated in bendamustine hydrochloride-resistant lymphoma cells (28), doxorubicin-resistant cervical cancer cells (29), and DDP-resistant NSCLC cells (11). This study also found that MGST1 was abnormally up-regulated in DDP-resistant NSCLC patients and cells. Furthermore, the DDP sensitivity of NSCLC cells was significantly improved after MGST1 ablation. This provides us with a novel understanding of DDP resistance mediated by MGST1 in NSCLC. 

Iron metabolism pathways are dysregulated to reduce iron accumulation for cancer proliferation (30). Ferroptosis induction is considered an effective treatment for cancer (31). It has been recognized that iron-dependent oxidative injury and subsequent lipid peroxidation exert critical roles in ferroptosis induction (32). Thus, promoting iron-dependent ROS release and oxidative stress may reinforce the antitumor effect of ferroptosis-related therapies. Of note, ferroptosis induction by ubiquitin-specific protease 7 (USP7) silencing could increase the DDP resistance of NSCLC cells (33). Another study also indicated that **α-**Hederin facilitated ferroptosis to overcome DDP chemoresistance in NSCLC cells (34). However, no publications exist about MGST1-mediated Fe2+ overload and DDP resistance in NSCLC. In the present study, we demonstrated that MGST1 depletion promoted ferroptosis by enhancing lipid peroxidation marker MDA level, ROS release, and Fe^2+^ accumulation, inhibiting antioxidant GSH production, as well as regulating expression of ferroptosis-related proteins GPX4, SLC7A11, and ACSL4. While ferroptosis inhibitor reversed shMGST1-mediated sensitization of NSCLC cells to DDP. These results suggested that ferroptosis induction was involved in MGST1 knockdown-induced DDP sensitivity of NSCLC cells. Targeting Fe^2+^ metabolism via MGST1 inhibition might be a new method for NSCLC treatment. These findings shed novel light on the knowledge of DDP resistance and MGST1-modulated Fe^2+^ metabolism. 

This study further suggested that MGST1 interacted with ALOX5 to inhibit its expression, which was an important mechanism responsible for ferroptosis suppression. Lipid peroxidation prefers to oxidize polyunsaturated fatty acids by ALOX family proteins and consequently causes membrane injury during ferroptosis (12). ALOX5 is one of the important members of the ALOX family, which is essential for ferroptosis of cancer cells. Liu *et al*. documented that ALOX5 deficiency facilitated bladder cancer development by promoting ferroptosis evasion (13). In this study, lower expression of ALOX5 and higher expression of MGST1 were found to be correlated with DDP resistance in NSCLC samples. Moreover, there was a negative correlation between ALOX5 and ALOX5 levels. The biological function of ALOX5 can be modulated by its binding proteins. As documented by Wenzel *et al*., phosphatidylethanolamine-binding protein 1 (PEBP1) is bound to ALOX15 to enhance ferroptosis of human airway epithelial cells (35). However, another study reported that MGST1 inhibited ALOX5 expression via a direct interaction, which restrained ferroptosis in pancreatic cancer cells (16). Consistent with the latter study, our data displayed that MGST1 protein bound to ALOX5 protein to restrain its expression, thus causing ferroptosis inhibition in NSCLC cells. Furthermore, exogenous overexpression of ALOX5 significantly weakened sh-*MGST1*-mediated ferroptosis and DDP sensitivity. These findings revealed that MGST1 interplayed with ALOX5 to repress ferroptosis, thus contributing to the DDP resistance of NSCLC cells. Therefore, we deduce that MGST1/ALOX5 axis-induced ferroptosis inhibition is fundamental in the emergence of DDP resistance in NSCLC cells, highlighting a potential therapeutic target to counteract DDP resistance.

There are several limitations in this study. First, there was a lack of sufficient patient samples to fully prove the correlation between abnormal expression of MGST1/ALOX5 and DDP resistance. Second, the possible regulatory mechanisms for the high expression of MGST1 in DDP-resistant NSCLC cells remain unclear. Future efforts will be focused on further validating the possibility of increasing DDP response in the clinic. Due to the more complicated expression and regulation in the human body, there is a long way to go before these findings are applied to the clinic.

## Conclusion

Our results demonstrated that high expression of MGST1 resulted in ferroptosis evasion via interplaying with ALOX5 protein, which consequently caused DDP resistance in NSCLC cells* in vitro *and *in vivo*. Targeting the MGST1/ALOX5 pathway might be a promising therapeutic strategy to conquer DDP resistance in NSCLC. 

## Declaration

We have not used any AI tools or technologies to prepare this manuscript.
